# Supplementary material for: Statistical Methods for Microrheology of Airway Mucus with Extreme Heterogeneity
Source: bioRxiv. 2023 Nov 21:2023.11.20.567244. Preprint. [Version 1] doi: 10.1101/2023.11.20.567244 (PMC10690152; doi:10.1101/2023.11.20.567244)
Supplement: 1 [file NIHPP2023.11.20.567244V1-supplement-1.pdf]

bioRxiv preprint doi: https://doi.org/10.1101/2023.11.20.567244; this version posted November 21, 2023. The copyright holder for this preprint (which was not certified by peer review) is the author/funder, who has granted bioRxiv a license to display the preprint in perpetuity. It is made available under aCC-BY 4.0 International license.

Supplemental Material

The fARMAs Denoising Method

For ease of presentation, we describe the method for a one-dimensional particle trajectory X(t), with extension to higher dimensions in [15]. The fARMA model [15] assumes that $X(t)$ is fractional Brownian motion with linear drift,

$$X(t) = \mu t + \sqrt{2D_\alpha} B_\alpha(t)$$

However, we observe not $X(t)$ but rather $Y_0, \dots, Y_N$, corresponding to noisy measurements of $X_n = X(n \cdot \tau)$, from which we must extract the pure entropic fluctuation signal. This approach to signal extraction was first proposed by Savin and Doyle [27] using the measurement model

$$\begin{aligned} Y_n &= \tilde{X}_n + \epsilon_n, \\ \tilde{X}_n &= \frac{1}{\tau}\int_0^\tau X(n \cdot \Delta t - s)\, ds, \end{aligned} \tag{12}$$

where ϵ_n is white noise representing "static" localization errors, and $\tilde{X}_n$ represents "dynamic" errors due to motion of the particle during the camera open aperture time τ. Static errors have the effect of raising the MSD of Y relative to that of X at the shortest timescales, whereas dynamic errors have the opposite effect. While theoretically appealing, Ling et al. [15] found the Savin-Doyle model lacking in flexibility to account for the complex interactions between signal and noise from one experimental lab to another. Instead, they propose an ARMA model (fARMA),

$$\begin{aligned} Y_n &= \tilde{X}_n + \epsilon_n, \\ \tilde{X}_n &= \theta \tilde{X}_{n-1} + (1 - \theta - \rho) X_n + \rho X_{n-1}, \end{aligned} \tag{13}$$

to which we have added the static noise term ϵ_n here and in [33] that we have found necessary for in-flake particles, illustrated in Figure 3 with synthetic data. We refer to model (13) as fARMAs.

bioRxiv preprint doi: https://doi.org/10.1101/2023.11.20.567244; this version posted November 21, 2023. The copyright holder for this preprint (which was not certified by peer review) is the author/funder, who has granted bioRxiv a license to display the preprint in perpetuity. It is made available under aCC-BY 4.0 International license.

Parameter Estimation

To estimate the parameters of the fARMAs model (13), it is convenient to transform to an unconstrained basis; this improves model convergence around the parameter endpoints in the standard basis. Since $\alpha \in (0,2)$, an intuitive unconstrained transformation is:

$$\phi_1 = \mathrm{logit}(\alpha; 0,2)$$

where the generalized logit transformation is given by:

$$\mathrm{logit}(x; L, U) = \log\left(\frac{z}{1-z}\right), \qquad z = \frac{x-L}{U-L}.$$

Similarly, the noise parameters are transformed using the logit transform: for the fARMAs model $\phi_2 = \mathrm{logit}(\rho; -1,1)$ and $\phi_3 = \mathrm{logit}(\theta; -1,1)$. The drift coefficients, μ, are inherently unrestrained, so the last parameter to transform is the scaling matrix Σ. The unconstrained parametrization for Σ is:

$$\lambda = \left(\log\left(tr(\Sigma)\right), \log(\Sigma_{11}/\Sigma_{22}), \mathrm{logit}(\kappa; -1,1)\right), \qquad \kappa = \frac{\Sigma_{12}}{\sqrt{\Sigma_{11}\Sigma_{22}}}.$$

Optimization is then done using maximum likelihood estimation (MLE). However, MLE requires numerical optimization of the likelihood function, which can be highly sensitive to the choice of initial value. To address this, we develop a predictor-corrector algorithm: the predictor calculates the LS estimates of (α, D_α), the corrector uses predictor estimates to initialize the fARMAs model.

Choosing a threshold for the Noise Floor

Beads that become embedded in mucosal flakes exhibit low mobility. At the lowest levels of mobility, $\alpha < 0.1$, fARMAs is unstable, especially in the estimation of D_α as shown with beads stuck to the glass plate in Figure 9.

bioRxiv preprint doi: https://doi.org/10.1101/2023.11.20.567244; this version posted November 21, 2023. The copyright holder for this preprint (which was not certified by peer review) is the author/funder, who has granted bioRxiv a license to display the preprint in perpetuity. It is made available under aCC-BY 4.0 International license.

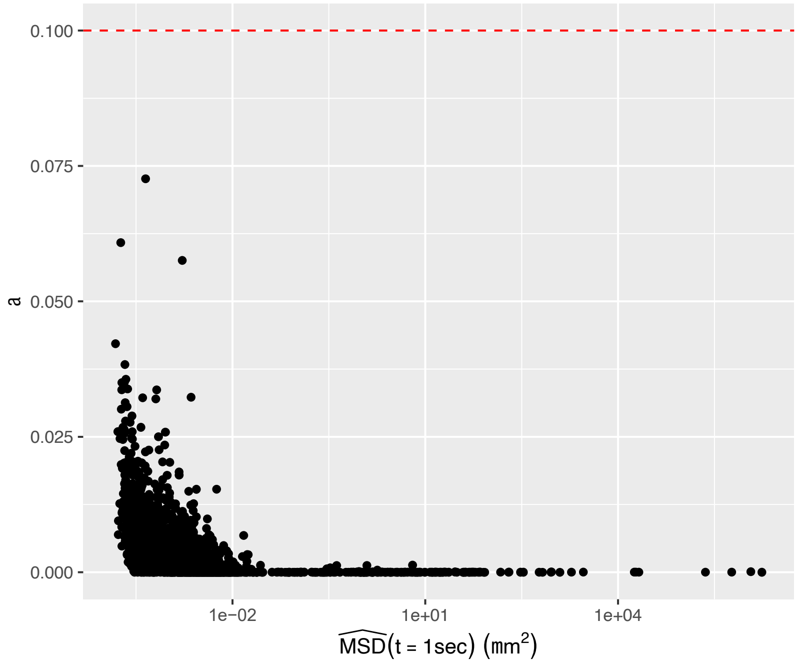

Figure 9: Plot of (α, Δ) values for stuck beads whose signals are purely noise. The broad range of Δ estimates, almost 10 orders of magnitude, reflect the instability in estimation of Δ when $\alpha < 0.1$. Based on this data, we impose the dashed red line $\alpha = 0.1$ as the noise floor cutoff.

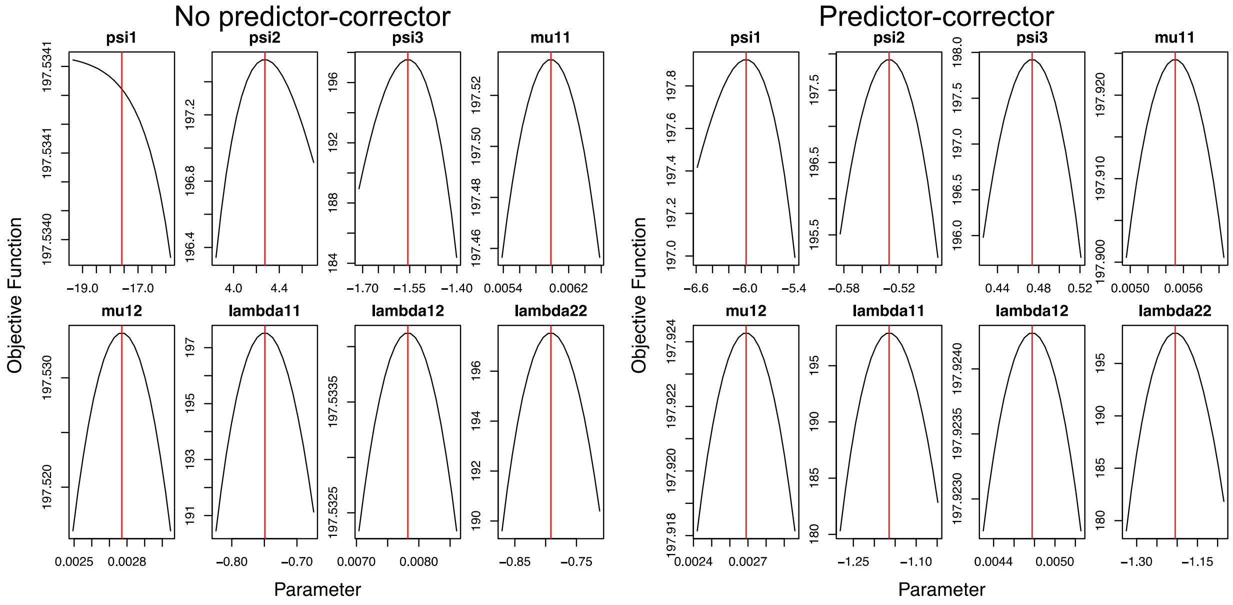

Figure 10: Convergence graph for optimization parameters for fARMAs. ψ_1 corresponds to α, $\psi_{2,3}$ are noise parameters, μ are the linear drift parameters in x and y, and λ are the scaling parameters.

Accuracy Comparison of fARMAs and LS on Truth Set Data

The accuracies of LS and fARMAs are tested using a set of synthetic truth sets, using $(\alpha, \widetilde{D}_\alpha)$ values consistent with experimental data. Two high-frequency error models are included in the

bioRxiv preprint doi: https://doi.org/10.1101/2023.11.20.567244; this version posted November 21, 2023. The copyright holder for this preprint (which was not certified by peer review) is the author/funder, who has granted bioRxiv a license to display the preprint in perpetuity. It is made available under aCC-BY 4.0 International license.

simulation: no error, and static and dynamic error. In addition, linear drift is added to all simulated trajectories. Six pairs of $(\alpha, \widetilde{D}_\alpha)$ are used to simulate trajectories: one for water, two for in-between water-like and flake-like, two flake-like, and one in the noise floor. One hundred trajectories are simulated for each set of (α, D_α) values: $\alpha = (0.999, 0.75, 0.5, 0.25, 0.15, 0.05)$, $D_\alpha = (0.43, 0.108, 0.0108, 0.0027, 0.0014, 0.00096)$ pulled from experimental data.

For each set of values one hundred trajectories are simulated using pure fBm and then fBm + static/dynamic noise. For both methods, constant linear drift is added. For X and Y the drift coefficient is chosen using the runif() function in R. This generates a random number from a uniform distribution, in our case between -0.025 and 0.025.

Trajectories are simulated from the fBm autocorrelation function, fbm_acf() in the **subdiff** package [37], and then converted to increments using the rnormtz() function in the **SuperGauss** package [38]. For the fBm + noise model, trajectories are simulated via the same process but using fsd_acf(), the Savin and Doyle error model autocorrelation function, instead of fbm_acf(). For all (α, D_α) pairs the value of $\tau = 0.5$ is used, while the signal to noise ratio changes: $SNR = (10, 5, 1, 0.1, 0.09, 0.08)$.

Once a trajectory is simulated, LS is used to get an estimate, which is used as the predictor step to be corrected by both fARMA and fARMAs models. To determine which model returns the most accurate estimate, the mean squared error, MSE, is computed between the fitted MSD and the empirical MSD between 1/60 and 10 seconds. Whichever model gives the smaller MSE is then used, and both the LS and fARMA(s) model's α and D_α values are recorded. Figure 11 shows the true values and mean estimates (left) and then error bars corresponding to twice the mean standard error for each (right).

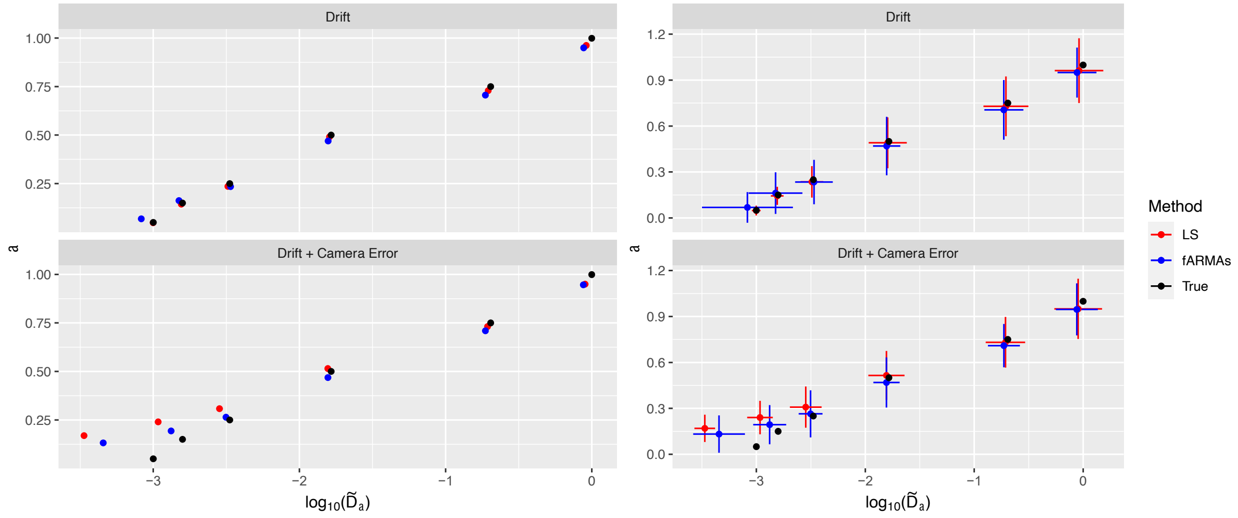

Figure 11: $(\alpha, \widetilde{D}_\alpha)$ estimates for each of the six bead pairs for the two error models: low frequency, and low plus high frequency. The left panel gives the estimates from LS and LS predictor-fARMAs corrector and the true values, while the right panel superimposes crosses consisting of two times the standard error in α and $\widetilde{D}_\alpha$.

bioRxiv preprint doi: https://doi.org/10.1101/2023.11.20.567244; this version posted November 21, 2023. The copyright holder for this preprint (which was not certified by peer review) is the author/funder, who has granted bioRxiv a license to display the preprint in perpetuity. It is made available under aCC-BY 4.0 International license.

Figure 11 shows that in cases of no high frequency noise or when α and $\widetilde{D}_\alpha$ are close to water values, LS and fARMAs give similar results. However, when there is high-frequency error in the signal and α- $\widetilde{D}_\alpha$ exhibit a flake-like signal, fARMAs corrects inaccuracy of LS when the LS predictor-fARMAs corrector method is used. For signals in the noise floor and with high-frequency error, both LS and LS-fARMAs are inaccurate.

Data Cleaning

Before trajectories are analyzed, the data is cleaned. First, we filter non-isolated beads -- those within five diameters of another bead at the initial time step. This is necessary as our classifier approach assumes beads are undergoing fluctuations solely due to medium fluctuations, whereas non-isolated beads transmit forces to one another [17, 18, 19], discussed further below. The second filter ensures trajectories contain the full increment time series, automatically enforced in our experimental lab. For data in this paper, results of data cleaning are given in the table below.

Sample	Total Beads	Proximity Filter	Length Filter	Total Left
1μm HBE + Calu3 60%	154	29	0	125
1μm HBE + Calu3 80%	137	2	0	135
1μm HBE + Calu3 90%	36	2	0	34
200nm HBE + Calu3 60%	17	0	0	17
200nm HBE + Calu3 80%	31	0	0	31
200nm HBE + Calu3 90%	32	0	0	32

Beads inside flakes, especially for 1 μm beads in the 60% solution, may be within five diameters of one another, an inherent effect of flake size relative to the beads. The fit of each bead time series to pure fractional Brownian motion (fBm) plus high and low frequency noise, and insertion of the MSD formula from the inferred fBm classifier parameters into the generalized Stokes-Einstein relation, assume the only forces acting on the bead are from fluctuations of the medium. However, beads in close proximity propagate forces between one another. This coupling is the basis for two-bead microrheology (cf. [1, 3, 17, 19, 20, 18]), which we do not apply here. For all remaining bead trajectories after the first two filters, we estimate the diffusion parameters, α and D_α. A final filter is then applied to bead trajectories indistinguishable from the noise floor of this experimental setup. The remaining bead data after these three filters is then analyzed.

Sample	Water-Like signals	Flake-Like signals	Noise Floor signals
1μm HBE + Calu3 60%	89	18	18
1μm HBE + Calu3 80%	101	23	11
1μm HBE + Calu3 90%	23	8	2

bioRxiv preprint doi: https://doi.org/10.1101/2023.11.20.567244; this version posted November 21, 2023. The copyright holder for this preprint (which was not certified by peer review) is the author/funder, who has granted bioRxiv a license to display the preprint in perpetuity. It is made available under aCC-BY 4.0 International license.

200nm HBE + Calu3 60%	0	6	11
200nm HBE + Calu3 80%	3	21	1
200nm HBE + Calu3 90%	0	0	30

Sample	Percent Water-Like	Percent Flake-Like	Percent Noise Floor
1μm HBE + Calu3 60%	71%	14%	14%
1μm HBE + Calu3 80%	74%	17%	8%
1μm HBE + Calu3 90%	69%	24%	6%
200nm HBE + Calu3 60%	0%	35%	64%
200nm HBE + Calu3 80%	12%	84%	4%
200nm HBE + Calu3 90%	0%	0%	100%
